# Bridging the Gap Between Knowledge and Practice: Contemporary Preventive Strategies in Modern Dental Care—A Cross-Sectional Survey of Practicing Dentists

**DOI:** 10.3390/jcm15135027

**Published:** 2026-06-27

**Authors:** Liana Beresescu, Alexandra Mihaela Stoica, Andrea Bors, Adina Simona Cosarca, Gabriela Felicia Beresescu, Alexandru Vlasa, Elena Stepco, Csilla Benedek

**Affiliations:** 1 Faculty of Dental Medicine, University of Medicine and Pharmacy, Science, and Technology George Emil Palade, 540139 Targu-Mures, Romania; liana.beresescu@umfst.ro (L.B.); andrea.bors@umfst.ro (A.B.); adina.cosarca@umfst.ro (A.S.C.); felicia.beresescu@umfst.ro (G.F.B.); alexandru.vlasa@umfst.ro (A.V.); csilla.benedek@umfst.ro (C.B.); 2 Faculty of Dental Medicine, The State University of Medicine and Pharmacy “Nicolae Testemitanu”, MD-2004 Chisinau, Moldova

**Keywords:** dental caries, primary prevention, risk assessment, minimally invasive surgical procedures, dentists, education, continuing

## Abstract

**Background/Objectives:** Although contemporary preventive concepts are well established in dentistry, their consistent integration into routine clinical practice remains uncertain. This study aimed to assess how preventive strategies are understood and applied in daily dental practice, and to explore the relationship between clinicians’ level of familiarity with preventive concepts and their implementation in patient care. **Materials and Methods:** A cross-sectional survey was conducted among 202 practicing dentists between October 2024 and May 2025 using a structured, anonymous questionnaire comprising 34 items. The instrument explored professional characteristics, knowledge of preventive concepts, clinical decision-making, use of fluoride-based interventions and minimally invasive approaches, and familiarity with risk-based systems. Data were analyzed using descriptive statistics and chi-square and Fisher’s exact tests (*p* < 0.05). **Results:** Most respondents reported moderate to high familiarity with contemporary preventive concepts, particularly remineralization and fluoride-based prevention. Preventive measures were commonly used; however, their implementation was often not structured. Formal caries risk assessment was routinely or often performed by 69.3% of clinicians, yet structured systems such as CAMBRA were routinely or often used by only 19.8%. Continuing professional education was significantly associated with greater use of preventive technologies (*p* = 0.018), and the use of structured risk assessment was associated with risk-based restorative decision-making (*p* = 0.041). **Conclusions:** Respondents reported a high level of familiarity with preventive concepts, but their application appeared inconsistent and frequently unstructured. These findings highlight a persistent gap between familiarity and implementation and point toward the need for clinically feasible, structured approaches that can support preventive decision-making in routine care.

## 1. Introduction

In daily clinical practice, dental caries continues to be frequently managed at a restorative stage, despite growing evidence that early lesions can often be controlled or, in some cases, even reversed through preventive approaches [1,2]. This reflects the ongoing transition in dentistry from a predominantly restorative approach toward a more preventive and minimally invasive model of care. In this context, dental caries is increasingly recognized as a dynamic, biofilm-mediated process that can be influenced by timely intervention. When detected early, the disease process can often be arrested and, in certain situations, partially reversed, reducing the need for surgical treatment and preserving tooth structure [3,4,5,6,7,8].

Today, preventive dentistry extends beyond routine oral hygiene instructions and fluoride recommendations and increasingly involves individualized, risk-based clinical decision-making. Prevention is approached as a personalized process based on a combination of risk assessment, dietary counseling, professional interventions, and minimally invasive techniques [9,10,11]. Contemporary clinical frameworks such as the International Caries Classification and Management System (ICCMS) and Caries Management by Risk Assessment (CAMBRA) have been developed to support this approach by aligning clinical decisions with lesion activity and patient risk profile [12,13,14].

However, translating these concepts into routine care remains inconsistent. Although these preventive approaches are widely discussed in the literature and continuing professional education, their implementation in everyday practice still appears variable [15,16]. In reality, clinical decision-making is rarely guided by evidence alone, but is influenced by a range of practical factors, including limited chairside time, patient expectations, financial considerations, and the availability of materials or training. Barriers such as reduced patient compliance and time constraints are frequently reported as limiting factors in the implementation of preventive strategies [17,18].

Although dental caries is largely preventable, it remains highly prevalent worldwide This observation suggests that a gap may still exist between awareness of preventive concepts and their consistent implementation in routine clinical practice [19,20]. Understanding how contemporary preventive strategies are applied in everyday clinical decision-making may help identify factors influencing their adoption. The aim of this study was to evaluate how contemporary preventive strategies are understood and applied in routine dental practice. Specifically, the study aimed to explore the relationship between clinicians’ level of familiarity with modern preventive approaches and their implementation in patient care, as well as to identify the main factors that may limit their consistent use, particularly regarding risk-based prevention, fluoride-based interventions, and minimally invasive techniques used in everyday clinical care.

## 2. Materials and Methods

This cross-sectional survey was conducted among practicing dentists between October 2024 and May 2025 using a structured, anonymous questionnaire designed to explore how preventive strategies are implemented in routine clinical practice (Supplementary File S1). The questionnaire included 34 items addressing demographic and professional characteristics, self-reported familiarity with contemporary preventive concepts, clinical decision-making related to prevention, use of fluoride-based interventions, application of minimally invasive approaches, and familiarity with risk-based prevention systems.

Participants were recruited through the professional communication channels of the Romanian College of Dentists. The questionnaire was distributed electronically to practicing dentists in Romania. Dentists actively involved in clinical practice were eligible to participate; no additional exclusion criteria were applied. A formal sample size calculation was not performed prior to study initiation. The study used a convenience sampling approach, and all eligible dentists who voluntarily responded to the questionnaire during the study period were included in the analysis. The questionnaire was distributed electronically using an online survey platform; participation was voluntary, informed consent was obtained from all participants, and no personal identifying data were collected. Due to the open distribution of the questionnaire through professional communication channels, the total number of dentists reached could not be determined; therefore, the response rate was not calculated.

The questionnaire was structured into five sections addressing: (1) professional background, (2) knowledge of preventive concepts, (3) preventive practices in daily clinical work, (4) use of contemporary preventive technologies, and (5) attitudes and perceived barriers to implementation. Its development was guided by current literature and contemporary preventive dentistry frameworks, particularly ICCMS and CAMBRA, as well as evidence-based caries management principles [12,13,14], with the aim of reflecting routine clinical decision-making rather than purely theoretical knowledge. The questionnaire was reviewed for clarity, relevance, and content validity by three clinicians with experience in preventive dentistry, and minor adjustments were made following their feedback to improve clarity and consistency prior to distribution. However, despite this expert review, no formal psychometric validation, reliability assessment, or internal consistency analysis was performed. Responses were collected using ordinal response categories. For descriptive analyses, selected adjacent categories were combined to facilitate interpretation of the findings and to avoid excessive fragmentation of responses across multiple categories. Specifically, “very familiar” and “moderately familiar” were grouped as moderate/high familiarity, while “routinely” and “often” were grouped as frequent implementation of preventive practices. These grouped categories were used to provide a simplified overview of response patterns and should not be interpreted as implying complete equivalence between the original response options.

Data were collected and subsequently analyzed to describe patterns of preventive practice and to explore the relationship between clinicians’ level of self-reported familiarity with preventive concepts and the implementation of preventive strategies in daily care. Most variables were categorical and were analyzed accordingly. Descriptive statistics were used to summarize participant characteristics and response distributions. Associations between categorical variables—such as professional experience, level of knowledge, and use of preventive approaches—were assessed using the chi-square test. Chi-square test assumptions were assessed, and Fisher’s exact test was used when expected cell counts were low. All statistical analyses were performed using IBM SPSS Statistics (version 26.0, IBM Corp., Armonk, NY, USA). A *p*-value < 0.05 was considered statistically significant. Given the exploratory nature of the study, the analysis focused on bivariate associations. No multivariable analyses were performed, and no adjustment for multiple comparisons was applied. Therefore, the observed associations should be interpreted as exploratory. Incomplete responses were excluded from analysis where applicable.

The study protocol was reviewed and approved by the Ethics Committee of Denta Aur Private Medical Center (approval no. 17/03.09.2024). All procedures were conducted in accordance with relevant ethical standards for research involving human participants.

## 3. Results

### 3.1. Participant Characteristics

A total of 202 dentists completed the questionnaire. The demographic and professional characteristics of the participants are summarized in Table 1.

Most respondents were under the age of 30 (57.4%), followed by those aged 30–40 years (21.8%), 41–50 years (16.8%), and over 50 years (4.0%). Female dentists represented most participants (76.2%), while male dentists accounted for 23.8%.

In terms of professional background, general dentistry was the main field of practice for 57.4% of respondents. The remaining participants reported working in various specialties (34.7%), while 7.9% practiced pediatric dentistry.

Most respondents had less than 5 years of clinical experience (63.4%). Dentists with 11–20 years of experience accounted for 13.9%, those with more than 20 years for 12.8%, and those with 6–10 years of experience for 9.9%.

Most participants worked in private practice settings (69.3%), while 19.8% reported working in both private and public sectors and 10.9% worked exclusively in public institutions. The majority practiced in urban areas (81.2%), followed by mixed urban–rural settings (13.8%) and rural areas (5.0%).

Continuing professional education was common among respondents, with 60.4% attending courses or conferences several times per year and an additional 32.7% attending at least once per year.

### 3.2. Knowledge of Contemporary Preventive Strategies

Overall, respondents demonstrated a high level of familiarity with preventive dentistry (Table 2). A total of 41.6% reported being very familiar with contemporary preventive strategies, while 46.5% reported moderate familiarity. Only a small proportion reported slight familiarity (9.9%) or no familiarity (2.0%).

Knowledge regarding the reversibility of early caries lesions was also high. Most respondents (92.1%) reported moderate or high familiarity with the concept of remineralization and its clinical relevance. Similarly, 94.1% reported moderate or high familiarity with the role of fluoride in caries prevention.

When asked about newer remineralization approaches, such as CPP-ACP and bioactive materials, 68.3% reported moderate or high familiarity. Familiarity with contemporary methods for early caries detection was reported as moderate or high by 72.3% of participants. A high level of familiarity with contemporary preventive concepts was observed across respondents.

### 3.3. Preventive Practices in Daily Clinical Work

Preventive measures were commonly incorporated into clinical practice, although their use varied across different interventions (Table 3).

Dietary counseling was routinely provided by 38.6% of dentists and often by 34.7%. Occasional use was reported by 19.8%, while 6.9% reported rarely providing dietary advice.

Assessment of individual caries risk prior to treatment planning was routinely performed by 36.6% of respondents and often performed by 32.7%. A further 19.8% reported occasional use, and 10.9% reported rarely performing formal risk assessment. When combining the categories “routinely” and “often”, formal caries risk assessment was performed by 69.3% of respondents.

When asked about the methods used for risk assessment, most dentists reported relying on clinical examination and professional judgment (91.1%). Patient history, including previous caries experience and oral hygiene habits, was used by 83.2% of respondents, while radiographic findings were considered by 72.3%. Clinical indices, such as DMFT or plaque index, were used by 33.7%, and structured risk assessment systems (e.g., CAMBRA or Cariogram) were used by 21.8% of respondents overall, although routine or frequent use was reported by only 19.8%. A small proportion (8.9%) reported not performing formal risk assessment.

Individualized oral hygiene instructions were routinely provided by 54.5% of dentists and often provided by 32.7%, with fewer respondents reporting occasional or rare use.

Fluoride toothpaste recommendation was part of routine practice for most respondents (81.2%), while 15.8% reported often recommending it. Only a small number reported occasional or rare recommendations.

Additional oral hygiene measures, such as dental floss, interdental brushes, or fluoride mouthwash, were routinely recommended by 42.6% of respondents and often recommended by 34.7%. The remaining respondents reported occasional or rare use of these measures.

Regarding restorative decision-making, 44.6% of dentists reported that their decision to initiate restorative treatment depended on individual caries risk and lesion activity. In contrast, 29.7% reported initiating restorative treatment only when cavitation into dentin was clinically evident. The remaining respondents reported using other decision thresholds.

### 3.4. Use of Contemporary Preventive Technologies

The use of structured preventive systems and contemporary technologies varied considerably, with particularly low adoption of risk-based caries management approaches. The results are summarized in Table 4. Risk-based systems such as CAMBRA were routinely used by 7.9% of dentists and often used by 11.9%, while the remaining respondents reported occasional, rare, or no use.

Pit and fissure sealants were widely used for preventive purposes. Routine use was reported by 52.5% of respondents, while 31.7% reported often using sealants. Occasional use was reported by 11.9%, and only 4.0% reported rare or no use.

Therapeutic use of sealants for the management of early non-cavitated lesions was less frequent. Routine use was reported by 29.7% of respondents, followed by frequent use (32.7%), occasional use (21.8%), and rare or no use (15.8%).

Professional topical fluoride applications were routinely used by 36.6% of respondents and often used by 34.7%, while the remaining respondents reported occasional, rare, or no use.

Resin infiltration techniques were routinely used by 19.8% of respondents and often used by 24.8%. More than half of respondents (55.4%) reported rare or no use of this approach.

Silver diamine fluoride was not reported to be routinely used; however, 17.8% of dentists reported using it often, while the remaining respondents reported occasional, rare, or no use.

CPP-ACP remineralization products were routinely recommended by 29.7% of respondents and often recommended by 28.7%, while the remaining respondents reported occasional, rare, or no use.

Probiotics were routinely or often recommended by 22.8% of respondents, while the remaining respondents reported occasional, rare, or no use.

Laser technologies were routinely or often used by 14.9% of respondents, while the remaining respondents reported occasional, rare, or no use.

### 3.5. Attitudes Toward Contemporary Preventive Strategies and Perceived Barriers

Participants’ attitudes toward contemporary preventive strategies and the perceived barriers to their implementation are summarized in Table 5. Most respondents reported feeling confident in managing early caries lesions using preventive approaches. High confidence was reported by 68.3% of dentists, while 27.7% reported moderate confidence.

Preventive dentistry was considered a high- or very high-priority by 84.2% of respondents.

Professional satisfaction related to preventive practice was reported as high by 62.4% of respondents, while 30.7% reported moderate satisfaction.

Interest in further training was high, with 79.2% expressing strong interest in additional education in preventive dentistry.

The most reported barriers were limited patient compliance (56.4%), lack of time (42.6%), and financial constraints (31.7%). Multiple responses were allowed.

### 3.6. Associations Between Professional Characteristics and Preventive Practices

Several significant associations were observed between professional characteristics and the use of preventive strategies in clinical practice as summarized in Table 6.

The use of structured caries risk assessment systems, such as CAMBRA, was significantly associated with years of clinical experience (*p* = 0.032). Dentists with fewer than 5 years of clinical experience reported higher use compared to those with more than 5 years of experience.

Frequent participation in continuing professional education was significantly associated with greater use of contemporary preventive technologies, including resin infiltration, remineralization products, and other preventive interventions (*p* = 0.018). Dentists who attended continuing education activities more frequently reported higher use of these technologies compared to those with less frequent participation.

A significant association was also observed between the use of structured caries risk assessment systems and restorative decision-making (*p* = 0.041). Dentists who reported using structured risk assessment systems were more likely to base restorative decisions on individual caries risk and lesion activity.

Higher levels of familiarity with contemporary preventive strategies were significantly associated with greater implementation of preventive technologies in routine clinical practice (*p* = 0.027). Respondents reporting higher familiarity were more likely to implement these technologies compared to those with lower levels of familiarity.

In addition, dentists who participated more frequently in continuing professional education reported significantly greater use of structured caries risk assessment systems (*p* = 0.022).

## 4. Discussion

The present findings provide a clinically relevant perspective on how contemporary preventive concepts are translated into routine dental practice. While a high level of familiarity with key principles—such as remineralization, fluoride-based prevention, and early caries detection—was consistently reported, their application in daily clinical decision-making appeared less structured. In many situations, preventive concepts seem to be accepted at a theoretical level but applied selectively depending on the realities of everyday clinical practice. These results suggest that the challenge in modern preventive dentistry may lie not in knowledge acquisition itself, but in its consistent integration into everyday care.

This observation reflects a situation frequently encountered in daily dentistry, where clinicians are often required to balance evidence-based recommendations with practical limitations such as limited chairside time, patient expectations, treatment costs, and workflow efficiency. In this context, translating preventive concepts into predictable and reproducible clinical protocols may be more difficult than simply understanding their theoretical background.

This pattern is in line with previous reports, which indicate that although minimally invasive and preventive approaches are widely accepted, their integration into routine workflows remains uneven [21,22,23]. Frameworks such as ICCMS and CAMBRA have been developed to support risk-based decision-making; however, their adoption in daily practice continues to be limited [24,25,26]. The present findings are consistent with this observation, particularly regarding the low use of structured risk assessment systems despite high levels of theoretical familiarity. Interestingly, many clinicians appeared to rely primarily on individual clinical judgment rather than formalized risk assessment models. These findings may suggest that factors beyond awareness influence the use of structured risk assessment systems. However, the present study did not directly evaluate the reasons underlying their adoption or non-adoption, and this interpretation should therefore be made with caution. In practice, approaches that are overly complex are unlikely to be adopted consistently, which highlights the importance of simplified, clinically applicable risk assessment tools that can be readily integrated into everyday workflows, as also suggested by recent efforts to develop streamlined, questionnaire-based models for caries risk assessment in specific populations [16,17,27,28].

The high level of awareness regarding remineralization and fluoride-based prevention reflects the strong emphasis placed on these concepts in both undergraduate education and continuing professional development. Current evidence supports their effectiveness in arresting or reversing early caries lesions when applied appropriately [29,30,31,32]. However, the results suggest that these strategies are not always implemented systematically, which may reduce their overall clinical impact.

An important finding is the predominance of intuitive, experience-based approaches in caries risk assessment. Most clinicians reported relying primarily on clinical examination and professional judgment, while structured systems such as CAMBRA or Cariogram were used less frequently. This reliance on personal clinical experience is understandable and remains an essential component of decision-making in dentistry. However, in the absence of standardized assessment methods, treatment decisions may become more variable between clinicians and clinical settings. Previous studies have shown that structured risk assessment models can improve the consistency of clinical decisions and support more individualized preventive strategies [33,34]. For this reason, practical and easy-to-use assessment tools may be particularly valuable in supporting preventive care without significantly increasing clinical workload.

The use of contemporary preventive technologies also varied considerably. While conventional measures, such as fluoride toothpaste and sealants, were widely adopted, newer approaches—including resin infiltration, silver diamine fluoride, and bioactive materials—were used less consistently. This variation likely reflects differences not only in training and material availability, but also in clinician confidence and familiarity with these newer approaches. Although these minimally invasive techniques are increasingly supported by clinical evidence [35,36,37,38,39,40,41], their incorporation into routine care appears to be gradual rather than uniform. The preventive interventions evaluated in the present study differ considerably with respect to their evidence base, indications, accessibility, and clinical applicability. Therefore, lower adoption of certain approaches should not necessarily be interpreted as a deficiency in clinical practice, but may reflect differences in training, availability, regulatory context, cost, or the specific clinical situations in which these interventions are indicated.

The observed association between continuing professional education and increased use of preventive strategies may suggest a relationship between professional development and the adoption of preventive approaches. However, given the cross-sectional design and the exploratory nature of the analysis, causality cannot be inferred and potential confounding factors cannot be excluded. Clinicians who reported more frequent participation in educational activities were more likely to implement contemporary preventive technologies and structured risk assessment systems. This observation is consistent with previous studies that reported similar associations between continuing professional education and the adoption of preventive approaches [42,43,44,45]. However, the direction and independence of this association cannot be determined from the present study. From a practical perspective, the barriers identified—particularly limited patient compliance, time constraints, and financial considerations—are consistent with those reported in the literature. Preventive care often requires sustained patient engagement and additional chairside time, which may not always align with the realities of daily practice. As a result, clinicians may prioritize restorative interventions, even when preventive alternatives are available. In addition, patient motivation and long-term adherence to preventive recommendations remain difficult to maintain in many clinical situations. These constraints highlight the importance of developing preventive strategies and tools that are not only evidence-based, but also feasible within the time and structural limitations of daily practice.

Despite these barriers, most respondents reported high levels of confidence and a strong interest in further training. This suggests that the observed gap is not primarily driven by resistance to preventive concepts, but rather by challenges in their practical implementation. Overall, the findings indicate that clinicians generally recognize the importance of preventive dentistry and appear open to integrating contemporary approaches when these are practical, accessible, and easy to incorporate into routine workflows. Simplifying clinical protocols and facilitating access to structured tools may support a more consistent integration of preventive strategies into routine care. In this context, future research should focus on the development and validation of simplified, population-adapted risk assessment tools that can support clinical decision-making across different patient groups.

The findings of this study should be interpreted considering certain limitations. The analysis was primarily based on bivariate associations, and potential confounding factors were not adjusted for, which may have influenced the observed relationships. In addition, no adjustment for multiple comparisons was applied. Therefore, some statistically significant associations may have occurred by chance and the findings should be interpreted cautiously. Consequently, the reported associations should be interpreted as exploratory and hypothesis-generating. In addition, the results were primarily reported as descriptive measures and bivariate associations. The use of a self-reported questionnaire may introduce response bias, and social desirability bias, as participants may overestimate their level of familiarity with preventive concepts or adherence to preventive practices. Furthermore, the reported preventive practices could not be independently verified and may not fully reflect actual clinical behavior. In addition, the questionnaire was not formally validated, and no psychometric validation, reliability assessment, or internal consistency analysis was performed. Because the questionnaire was intended as a study-specific exploratory instrument rather than a psychometric measurement tool, the findings should be interpreted as descriptive estimates of self-reported familiarity and preventive practices rather than objective measures of knowledge or clinical competence. Therefore, the precision and reproducibility of these self-reported measures remain uncertain and should be interpreted with caution. Furthermore, the voluntary nature of participation may have introduced self-selection bias, as dentists with a greater interest in preventive dentistry may have been more likely to complete the questionnaire. The open distribution of the questionnaire did not allow for the calculation of a precise response rate, and the sample was predominantly composed of younger practitioners, which may limit the generalizability of the findings. Therefore, the findings should not be interpreted as representative of all dentists practicing in Romania. The demographic profile of the respondents may have influenced the observed patterns of familiarity with preventive concepts and their reported implementation. At the same time, the inclusion of a younger population may also offer insight into how contemporary preventive concepts are currently reflected in more recent dental education and early clinical practice.

## 5. Conclusions

The findings of this study indicate that, although respondents reported a high level of familiarity with contemporary preventive concepts, their consistent integration into routine clinical practice remains limited and often unstructured. The results suggest a persistent gap between awareness of preventive strategies and their implementation in everyday clinical care. From a clinical perspective, improving the integration of structured risk assessment tools and facilitating the adoption of simplified preventive protocols may support a more consistent use of minimally invasive strategies in daily care. An association was observed between continuing professional education and greater use of preventive approaches; however, the direction of this relationship requires further investigation. These findings support the need for clinically applicable, evidence-based frameworks that can facilitate the integration of preventive strategies into routine clinical practice, in line with current priorities in minimally invasive and preventive dental care.

## Figures and Tables

**Table 1:** Participant characteristics (*n* = 202).

Variable	*n*	%
Age < 30 years	116	57.4
Age 30–40 years	44	21.8
Age 41–50 years	34	16.8
Age > 50 years	8	4.0
Female	154	76.2
Male	48	23.8
Private practice	140	69.3
Public practice	22	10.9
Both sectors	40	19.8

**Table 2:** Knowledge of preventive concepts.

Variable	Moderate/High Familiarity (%)
Preventive strategies	88.1
Remineralization	92.1
Fluoride’s role	94.1
Bioactive materials/CPP-ACP	68.3
Early caries detection methods	72.3

**Table 3:** Routine and frequent use of preventive practices in daily clinical work.

Practice	Routinely (%)	Often (%)
Risk assessment	36.6	32.7
Oral hygiene instruction	54.5	32.7
Fluoride toothpaste recommendation	81.2	15.8

**Table 4:** Reported use of contemporary preventive technologies in clinical practice.

Technology	Routinely (%)	Often (%)	Rarely/Never (%)
CAMBRA	7.9	11.9	80.2
Sealants (preventive)	52.5	31.7	15.8
Resin infiltration	19.8	24.8	55.4
Silver diamine fluoride	0	17.8	82.2
CPP-ACP	29.7	28.7	41.6
Probiotics	0	22.8	77.2
Laser technologies	0	14.9	85.1

**Table 5:** Attitudes and perceived barriers.

Variable	%
High confidence	68.3
Moderate confidence	27.7
High interest in training	79.2
Patient compliance barrier	56.4
Time barrier	42.6
Financial barrier	31.7

**Table 6:** Significant associations between professional characteristics and preventive practices.

Association	Group 1	*n*/*N* (%)	Group 2	*n*/*N* (%)	*p*-Value
CAMBRA use vs. years of experience	≤5 years of experience	32/128 (25.0)	>5 years of experience	8/74 (10.8)	0.032
Technology use vs. continuing education	Several times per year	58/122 (47.5)	Once per year/rarely/never	24/80 (30.0)	0.018
Risk/activity-based restorative threshold vs. CAMBRA use	Routine/frequent CAMBRA use	24/40 (60.0)	Occasional/rare/never CAMBRA use	66/162 (40.7)	0.041
Technology use vs. familiarity with preventive concepts	Moderate/high familiarity	78/178 (43.8)	Slight/no familiarity	4/24 (16.7)	0.027
CAMBRA use vs. continuing education	Several times per year	31/122 (25.4)	Once per year/rarely/never	9/80 (11.3)	0.022

## Data Availability

The data presented in this study are available on reasonable request from the corresponding author. The data are not publicly available due to privacy and ethical considerations.

## Supplementary Materials

The following supporting information can be downloaded at: https://www.mdpi.com/article/10.3390/jcm15135027/s1, Supplementary File S1 (Questionnaire on Preventive Strategies in Dental Practice).
