# Supplementary material for: Bridging the Gap Between Knowledge and Practice: Contemporary Preventive Strategies in Modern Dental Care—A Cross-Sectional Survey of Practicing Dentists
Source: J Clin Med. 2026 Jun 27;15(13):5027. doi: 10.3390/jcm15135027 (PMC13363045; doi:10.3390/jcm15135027)
Supplement: Supplementary file 1 [file jcm-15-05027-s001.zip › jcm-4350916-supplementary.pdf]

Supplementary Material

Questionnaire: Clinical Implementation of Contemporary Preventive Strategies in Dental Practice

---

Section 1 – Professional Background

1. Age

- Under 30 years
- 30–40 years
- 41–50 years
- Over 50 years

2. Sex

- Male
- Female

3. Field of practice (primary clinical activity):

• General dentistry
• Pediatric dentistry
• Other specialty (please specify): __________

4. Years of clinical experience

- 0–5 years
- 6–10 years
- 11–20 years
- Over 20 years

5. Professional and academic status

- General dentist
- Specialist dentist
- Senior specialist / consultant
- University staff without PhD
- University staff with PhD

6. Type of practice

- Private
- Public
- Both

7. Practice location

- Urban
- Rural
- Both

8. Participation in continuing education (courses, conferences, workshops)

- Frequently (several times per year)
- Occasionally (once per year)
- Rarely
- Never

Section 2 – Knowledge of Preventive Concepts

9. Overall, how familiar are you with contemporary preventive strategies used in modern dental practice?

- Very familiar
- Moderately familiar
- Slightly familiar
- Not familiar

10. Are you aware that early caries lesions can be arrested or reversed through remineralisation?

- Yes, very familiar with this concept
- Yes, moderately familiar
- Slightly familiar
- Not familiar

11. How familiar are you with the remineralising role of fluoride in managing early caries lesions?

- Very familiar
- Moderately familiar
- Slightly familiar
- Not familiar

12. How familiar are you with newer remineralisation approaches other than fluoride (e.g., CPP-ACP, bioactive materials, resin infiltration)?

- Very familiar
- Moderately familiar
- Slightly familiar

- Not familiar

13. How familiar are you with contemporary methods for early caries detection?

- Very familiar
- Moderately familiar
- Slightly familiar
- Not familiar

Section 3 – Preventive Practices in Daily Clinical Work

14. How often do you provide dietary counselling as part of individualized preventive care?

- Routinely
- Often
- Occasionally
- Rarely
- Never

15. How often do you assess individual caries risk before deciding preventive or restorative treatment?

- Routinely
- Often
- Occasionally
- Rarely
- Never

16. How do you assess individual caries risk in your clinical practice? (Select all that apply)

- Based on clinical examination and professional judgment
- Based on patient history (past caries experience, diet, hygiene habits)
- Based on radiographic findings
- Using clinical indices (e.g., DMFT, plaque index)
- Using structured risk assessment systems (e.g., CAMBRA, Cariogram)
- I do not perform formal caries risk assessment

17. At what stage do you typically initiate restorative treatment for primary caries lesions under routine clinical conditions?

• Only when cavitation into dentin is clinically evident
• When cavitation limited to enamel is present
• At the stage of non-cavitated enamel lesions (initial lesions)

• Decision depends on individual caries risk and lesion activity
• I do not follow a consistent threshold

18. How often do you provide individualized oral hygiene instructions as part of preventive care?

- Routinely
- Often
- Occasionally
- Rarely
- Never

19. How often do you recommend fluoride toothpaste as part of caries prevention?

- Routinely
- Often
- Occasionally
- Rarely
- Never

20. How often do you recommend adjunctive oral hygiene measures (floss, interdental brushes, fluoride mouthwash)?

- Routinely
- Often
- Occasionally
- Rarely
- Never

Section 4 – Use of Contemporary Preventive Technologies

21. How often do you use risk-based caries management systems such as CAMBRA?

- Routinely
- Often
- Occasionally
- Rarely
- Never
- Not familiar with this concept

22. How often do you use pit and fissure sealants for caries prevention?

- Routinely
- Often
- Occasionally

- Rarely
- Never
- Not familiar with this technique

23. How often do you use sealants in the management of early non-cavitated caries lesions?

- Routinely
- Often
- Occasionally
- Rarely
- Never
- Not familiar with this technique

24. How often do you apply professional topical fluoride treatments (e.g., fluoride varnish, gel, or foam) as part of preventive dental care?

- Routinely
- Often
- Occasionally
- Rarely
- Never
- Not familiar with this technique

25. How often do you use resin infiltration (ICON) in clinical practice?

- Routinely
- Often
- Occasionally
- Rarely
- Never
- Not familiar with this technique

26. How often do you use silver diamine fluoride (SDF) in clinical practice?

- Routinely
- Often
- Occasionally
- Rarely
- Never
- Not familiar with this technique

27. How often do you recommend remineralisation products such as CPP-ACP or similar calcium-based agents?

- Routinely
- Often
- Occasionally
- Rarely
- Never

- Not familiar with this technique

28. How often do you recommend probiotics as an adjunct to preventive oral care?

- Routinely
- Often
- Occasionally
- Rarely
- Never
- Not familiar with this technique

29. How often do you use laser technologies as an adjunct to preventive or minimally invasive dental procedures?

- Routinely
- Often
- Occasionally
- Rarely
- Never
- Not familiar with this technique

Section 5 – Attitudes and Barriers

30. How confident are you in your ability to manage early caries lesions using preventive, non-invasive approaches?

• Very confident
• Moderately confident
• Slightly confident
• Not confident

31. What barriers, if any, limit your implementation of preventive dentistry in daily practice? (Select all that apply)

- Lack of time
- Limited patient compliance
- Lack of patient interest or motivation
- Financial constraints
- Lack of training
- Lack of materials or equipment
- No barriers

32. To what extent is preventive dentistry a priority in your daily clinical practice?

- Very high priority
- High priority
- Moderate priority

- Low priority
- Not a priority

33. To what extent does practicing preventive dentistry contribute to your professional satisfaction?

- Significantly
- Moderately
- Slightly
- Not at all

34. How interested are you in further training in preventive dentistry?

- Very interested
- Moderately interested
- Slightly interested
- Not interested
